# Interleukin-10 improves stroke outcome by controlling the detrimental Interleukin-17A response

**DOI:** 10.1186/s12974-021-02316-7

**Published:** 2021-11-13

**Authors:** Marius Piepke, Bettina H. Clausen, Peter Ludewig, Jonas H. Vienhues, Tanja Bedke, Ehsan Javidi, Björn Rissiek, Larissa Jank, Leonie Brockmann, Inga Sandrock, Karoline Degenhardt, Alina Jander, Vanessa Roth, Ines S. Schädlich, Immo Prinz, Richard A. Flavell, Yasushi Kobayashi, Thomas Renné, Christian Gerloff, Samuel Huber, Tim Magnus, Mathias Gelderblom

**Affiliations:** 1 Department of Neurology, University Medical Center Hamburg-Eppendorf, Martinistraße 52, 20246 Hamburg, Germany; 2 Department of Neurobiology Research, Institute of Molecular Medicine, University of Southern Denmark, Odense, Denmark; 3 I. Medizinische Klinik, University Medical Center Hamburg-Eppendorf, Hamburg, Germany; 4 Institute of Immunology, Hannover Medical School, Hannover, Germany; 5 Institute of Systems Immunology, University Medical Center Hamburg-Eppendorf, Hamburg, Germany; 6 Department of Immunobiology, The Howard Hughes Medical Institute, Yale School of Medicine, New Haven, CT USA; 7 Institute of Clinical Chemistry and Laboratory Medicine, University Medical Center Hamburg-Eppendorf, Hamburg, Germany

**Keywords:** Stroke, Ischemia, Inflammation, T cells, Interleukin-10, Interleukin-17

## Abstract

**Background:**

Lymphocytes have dichotomous functions in ischemic stroke. Regulatory T cells are protective, while IL-17A from innate lymphocytes promotes the infarct growth. With recent advances of T cell-subtype specific transgenic mouse models it now has become possible to study the complex interplay of T cell subpopulations in ischemic stroke.

**Methods:**

In a murine model of experimental stroke we analyzed the effects of IL-10 on the functional outcome for up to 14 days post-ischemia and defined the source of IL-10 in ischemic brains based on immunohistochemistry, flow cytometry, and bone-marrow chimeric mice. We used neutralizing IL-17A antibodies, intrathecal IL-10 injections, and transgenic mouse models which harbor a deletion of the IL-10R on distinct T cell subpopulations to further explore the interplay between IL-10 and IL-17A pathways in the ischemic brain.

**Results:**

We demonstrate that IL-10 deficient mice exhibit significantly increased infarct sizes on days 3 and 7 and enlarged brain atrophy and impaired neurological outcome on day 14 following tMCAO. In ischemic brains IL-10 producing immune cells included regulatory T cells, macrophages, and microglia. Neutralization of IL-17A following stroke reversed the worse outcome in IL-10 deficient mice and intracerebral treatment with recombinant IL-10 revealed that IL-10 controlled IL-17A positive lymphocytes in ischemic brains. Importantly, IL-10 acted differentially on αβ and γδ T cells. IL-17A producing CD4^+^ αβ T cells were directly controlled via their IL-10-receptor (IL-10R), whereas IL-10 by itself had no direct effect on the IL-17A production in γδ T cells. The control of the IL-17A production in γδ T cells depended on an intact IL10R signaling in regulatory T cells (Tregs).

**Conclusions:**

Taken together, our data indicate a key function of IL-10 in restricting the detrimental IL-17A-signaling in stroke and further supports that IL-17A is a therapeutic opportunity for stroke treatment.

**Supplementary Information:**

The online version contains supplementary material available at 10.1186/s12974-021-02316-7.

## Background

Worldwide, stroke is one of the leading causes of mortality and sustained disability, with 5.5 million deaths and 11 million disability-adjusted life-years in 2016 [1].

Inflammation is a key component of stroke pathophysiology, with dichotomous effects on the affected tissue. In the early stages, highly conserved pro-inflammatory pathways lead to a worsening of the initial tissue damage, whereas protective mechanisms are involved in tissue repair and ischemic tolerance in the long term [2].

Among the inflammatory cascades, which drive the early sterile immune response, IL-17A holds an important role. Following stroke, IL-17A is mainly produced by innate-like γδ T cells. By driving the expression of neutrophil attracting chemokines [e.g., C-X-C motif chemokines (CXCL-1)], IL-17A is crucial for the amplification of the local inflammatory response in the first hours, resulting in a worsened neurological outcome [3, 4]. However, IL-17A levels and numbers of infiltrating neutrophils already decrease 4 days after ischemia [5]. Immunological mechanisms that limit the IL-17A-dependent inflammatory response are largely unknown. Among potential anti-inflammatory pathways, IL-10 holds a prominent role, due to its strong immunomodulatory and neuroprotective effects [6, 7]. In stroke, IL-10 has been shown to negatively regulate proinflammatory cytokines (e.g., IFN-γ, TNF-α), and antigen-presenting cells, including microglia [6]. Although IL-10 and IL-17A have opposing effects on stroke outcome, it is largely unknown whether these two cytokines interact. Studies from models of autoimmunity [8, 9] indicate that regulatory CD4^+^ T cells and IL-10 represent a fundamental mechanism for regulating IL-17A. It has been shown that IL-10 can directly inhibit the IL-17A production of CD4^+^ effector T cells [8]. Interestingly, disruption of IL-10 signaling on CD4^+^ Tregs [10] and T regulatory type 1 cells (T_r_1) [11] also reduced their capacity to suppress the Th17 immune response which suggests an indirect suppression via an autocrine feed-forward loop.

Compared to the regulation of αβ T cells less is known about IL-10 effects on γδ T cells. However, Park et al. [12] suggested that Treg cell-derived IL-10 directly suppresses TCRγδ^+^ T cells by limiting their proliferation and IL-17A production in a model of inflammatory bowel disease.

The goal of this study was to analyze the role of the IL-10 signaling for the control of the detrimental IL-17A response in stroke. Our data show that IL-10 plays a key role in regulating IL-17A production in αβ and γδ T cells in the ischemic brain. Here, IL-10 acts differentially as CD4^+^ T cells are directly controlled, whereas intact IL-10R signaling on Tregs is required to suppress the IL-17A production in γδ T cells.

## Materials and methods

### Animals

All animal experiments were approved by local animal care committees (Behörde für Justiz und Verbraucherschutz der Freien und Hansestadt Hamburg; Lebensmittelsicherheit und Veterinärwesen, Hamburg, Germany). Experiments were conducted in accordance with the Guide for the Care and Use of Laboratory Animals published by the US National Institutes of Health (publication No. 83-123, revised 1996). All procedures were performed in accordance with the ARRIVE guidelines (Animal Research: Reporting of In Vivo Experiments). The number of mice required for assessing statistical significance of pre-specified effects was estimated by power analysis based on preliminary results and previous experience with the models used in our laboratory [4].

10–18-week-old male C57BL/6 mice provided by the animal facility of the University Medical Center Hamburg-Eppendorf were studied. All mice were backcrossed on a C57BL/6 background for > 10 generations. To evaluate effects on a global IL-10 deficiency and to perform bone-marrow transplantation we used *Il10*^*−/−*^ mice and respective wild-type (WT) littermate controls. Subsets of Tregs were studied in male double reporter *Foxp3-IRES-mRFP (FIR)* × *Il10-IRES-GFP-enhanced reporter (tiger)* mice. To deplete the IL-10R on CD4^+^, Foxp3^+^, or γδ T cells we took advantage of the Cre-loxP system and crossed *CD4*^*Cre*^, *Foxp3*^*Cre*^, and *TCRδ*^*CreER*^ with *Il10Ra*^*fl/fl*^ mice, respectively. *CD4*^*Cre*^ [13], *Foxp3*^*Cre*^ [14], *Il10Ra*^*fl/fl*^ mice [15], *FIR (Foxp3-IRES-mRFP)* [16], and tiger (*Il10-IRES-GFP-enhanced*) [17] reporter mice were kindly provided by S. Huber and are described elsewhere. *TCRδ*^*CreER*^ [18] mice were kindly provided by I. Prinz and are described elsewhere. For the induction of Cre-recombinase *TCRδ*^*CreER*^ animals were treated three times with 2 mg Tamoxifen within 5 days at the age of 8–10 weeks. Two weeks later, the animals were used for experiments. CD45.1^+^ WT and *Il10*^*−/−*^ mice were obtained from the Jackson Laboratory. For all animal experiments, mice were randomized and coded by an independent researcher to achieve blinding of the experiments. Age-matched male WT littermates served as controls. To reduce the variability of our outcome parameters (neurological outcome, inflammatory response) caused by sex differences and to thereby decrease group sizes, only male mice were used throughout the study. Recent studies have demonstrated profound effects of sex-differences on infarct sizes and the inflammatory response. To validate our studies in females, further studies are required.

### Transient middle cerebral artery occlusion (tMCAO)

We randomized all mice and conducted transient middle cerebral artery occlusion (tMCAO), using 6–0 nylon monofilament with silicone-coated tips (Doccol Corp.) as described previously [4]. Mice were anesthetized and monitored for heart rate, respiratory rate, oxygen saturation, and rectal body temperature. During filament-occlusion, cerebral blood flow was measured by laser-Doppler in the ipsi- and contralateral middle cerebral artery territory. Only mice with a reduction of > 80% in regional cerebral blood flow in the ipsilateral compared to the contralateral hemisphere were included. After 45 min, the monofilament was retracted to allow reperfusion for 3, 7, or 14 days. In the sham group, carotid arteries were visualized without insertion of the filament. Following surgery, animals were kept on heating pads and sterile saline was given via subcutaneous injection for rehydration. Exclusion criteria were defined in a score, which included weight loss, general condition, spontaneous behavior, and impairment of wound healing. We anesthetized all mice (20 to 25 g, 12 weeks; FTH, University Medical Center Hamburg-Eppendorf) using isoflurane 1% to 2% v/v oxygen. For analgesia we injected buprenorphine 0.03 mg/kg BW intraperitoneally every 12 h for 24 h. Mice were excluded from the study if they (i) died during the surgical procedure, (ii) experienced subarachnoid hemorrhage, (iii) had a Bederson score less than one after reawakening, or (iv) due to < 80% reduction in regional cerebral blood flow during MCAO.

### Antibody treatment

Animals were treated intravenously with 500 μg of mouse monoclonal anti-murine IL-17A antibody (Clone MM17F3; 16.6 mg/kg of body weight) or with 500 μg of isotype-control antibody (IgG1, BioXCell, MOPC-21, BE0083) 3 h and 3 days after reperfusion.

### Intracerebroventricular injections

Within 3 h after induction of MCA occlusion we performed an intracerebral injection of 200 ng control vehicle [bovine serum albumin (BSA) in 2 µl artificial cerebrospinal fluid consisting of mM: 126 NaCl; 2.5 KCl; 1.2 NaH_2_PO_4_; 1.3 MgCl_2_ and 2.4 CaCl_2_ at pH 7.4] or 200 ng IL-10 [recombinant mouse protein (rmIL-10 (carrier-free), Biolegend #575802) in 2 µl artificial cerebrospinal fluid].

Intracerebral injections were performed as previously described [19]: briefly, mice were anesthetized with isoflurane (4% for induction; 2.5% for maintenance) in 100% oxygen. The mice were placed in a stereotactic frame (Stoelting, 51500U) and a 1 cm long incision of the scalp was made over the midline. A cranial burr hole (0.9 mm) was drilled 2.2 mm lateral to the midline and 0.2 mm anterior to the bregma. A 10 µl Hamilton syringe (Hamilton, 1701RN) was connected to a 26-gauge needle (Hamilton, 26G, Point Style 4, 12°) and inserted into a motorized stereotaxic injector [Stoelting, integrated stereotaxic injector (ISI)]. The 26-gauge needle was then slowly introduced 3.7 mm deep into the left hemisphere and 2 μl of IL-10 or control vehicle was infused at a rate of 0.5 μl/min. The needle was left in place for another 10 min and then slowly withdrawn. Then a suture for the scalp incision was made. Animals recovered for a period of 60 min under a heating lamp with free access to food and water.

### Scoring

Following stroke induction, we repeatedly scored mice using the Bederson score on a scale from 0–5. This was done immediately after reawakening and every day until sacrifice. Bederson score: 0 no deficit, 1 preferential turning, 2 circlings, 3 longitudinal rollings, 4 no movement, 5 death. In addition, a composite neurological score was performed on 14 days after tMCAO, adapted from Orsini et al. [20]: scores range from 0 (healthy) to 59 (the worst performance in all categories) and represent the sum of the results of all categories. These include general deficits: hair (0–2), eyes (0–3), ears (0–2), posture (0–3), spontaneous activity (0–3), and focal deficits: circling (0–5), gait (0–4), body symmetry (0–3), climbing on a surface held at 45° (0–4), forelimb symmetry (0–4), compulsory circling (0–3), whisker response to a light touch (0–4), gripping (0–3), beam walking (0–9), balance on a beam (0–3), and balance on a round stick (0–4).

### Analysis of infarct size by triphenyltetrazolium chloride staining and magnetic resonance imaging (MRI)

The infarct size was analyzed 7 days post-reperfusion by harvesting brains and cutting them into 1 mm slices (Braintree Scientific, 1 mm) followed by vital staining using 2% (wt/vol) 2,3,5-triphenyl-2-hydroxy-tetrazolium chloride in phosphate buffer. We determined infarct volumes in a blinded fashion using NIH ImageJ software.

MRI comprised T2-weighted imaging and was performed on a dedicated 7T MR small animal imaging system (ClinScan; Bruker) 3 and 14 day post-reperfusion.

### Bone-marrow chimeras

For the generation of bone-marrow chimeras WT or *Il10*^*−/−*^ mice were reconstituted with either *Il10*^*−/−*^ or WT bone marrow, respectively. 6–8-week-old male recipients (CD45.2^+^) were exposed to whole-body irradiation (9 Gy; 1 Gy min^−1^) using a cesium-137 gamma irradiator (BIOBEAM 2000, Leipzig, Germany), as described before [21]. After 24 h, mice were reconstituted with bone-marrow cells derived from tibiae and femurs from CD45.2^+^ *Il10*^*−/−*^ or CD45.1^+^ WT mice, respectively. Every recipient received 10^7^ bone-marrow cells intravenously. We assessed reconstitution and distribution between CD45.1 and CD45.2 cells by FACS analysis of peripheral blood cells of recipient mice 6 weeks after grafting. tMCAO was induced in recipient mice 6 weeks after transplantation.

### Cell sorting

Immune cells were sorted using a BD FACS Aria IIIu and collected in RPMI with 25% fetal calf serum.

### RNA isolation and quantitative real-time polymerase chain reaction

We isolated RNA from brain tissue at indicated timepoints following tMCAO. Hemispheres were separated and homogenized in TRIzol Reagent (1 ml per 100 mg tissue), chloroform was added, samples were centrifuged at 12,000×*g* for 15 min at 4 °C and the upper aqueous phase was collected. RNA was precipitated by the addition of isopropyl alcohol, washed and dissolved in TE-Buffer. We isolated total RNA from cells using QIA-Shredder spin columns and the RNeasy Micro Kit (QIAGEN) and transcribed complementary DNA using Maxima First Strand cDNA Synthesis Kit for RT-qPCR (Fermentas). Real-time PCR primers were obtained from Applied Biosystems (Carlsbad, CA): Il6: Mm00446190_m1; Il23: Mm00518984_m1; Il1b: Mm00434228_m1; Tgfb: Mm01178820_m1; Cxcl1 Mm00433859_m1; Mmp3: Mm00442991_m1; BActin: Mm00607939_s1, Sdha: Mm01352366_m1. Probe mixtures were purchased from Fermentas (Waltham, MA). The relative gene expression was calculated using the ΔΔCt method and the samples were normalized to the control population and the expression of Sdha or β-Actin. Samples were randomized and coded by an independent researcher, so experiments were carried out blindly.

### Antibodies and flow cytometry

Cell types were analyzed by flow cytometry, as previously described [4]. Briefly, mice were euthanized and perfused with phosphate-buffered saline. Only ipsilesional hemispheres were dissected, digested for 30 min at 37 °C (1 mg/ml collagenase (Roche), 0.1 mg/ml DNAse I (Roche) in DMEM) and pressed through a cell strainer. Cells were incubated with standard erythrocyte lysis buffer on ice and separated from myelin and debris by Percoll gradient (GE Healthcare) centrifugation, followed by surface staining. In case of further intracellular staining T cells were stimulated with phorbol 12-myristate 13-acetate (100 ng/ml; Sigma-Aldrich) and ionomycin (1 μg/ml; Sigma-Aldrich) in the presence of brefeldin A (3 μg/ml; eBioscience) at 37 °C in 10% CO_2_ for 4 h before surface staining. To achieve fixation, permeabilization and intracellular staining True-Nuclear™ Transcription Factor Buffer Set (Biolegend, #424401) was used according to the manufacturer’s instructions. Mouse antibodies were as follows: from Biolegend CD45.2 (104; #109805), CD45.1 (A20, #110707), B220 (RA3-6B2, #103245, #103237), CD3 (145-2C11, #100308; 17A2, #100228), NK1.1 (PK136, #108745, #108713), TCR-γδ (GL-3, #118118), CD4 (RM4-5, #100553, #100551), CD8 (53.6-7, #100732, #100741, #100722), CD49b (HMα2, #103506), CD11b (M1/70, #101216), MHC II (M5/ 114.15.2; #107605), Ly6g (1 A8; #127627), F4/80 (BM8, # 123133), Lag-3 (C9B7W, #125210), PD-1 (29F.1A12, #135220), IFN-γ (XMG1.2, #505815, #505839), CD210 (1B1.3a, #112705); from eBioscience CD45 (30-F11, #47-0451-82, #56-0451-82), CD19 (eBio1D3, #25-0193-81), CD11c (N 418, #25-0114-82), TIGIT (GIGD7, #46-9501-82) and IL-17A (eBio17B1, #17-7177-81); from BD Bioscience CD4 (GK1.5, #563790) and RORγT (Q31-378, #562607). TruCount tubes (BD Bioscience), containing fluorescence beads were used to determine the total cell counts per hemisphere to compare the absolute number of IL-17A^+^ infiltrating CD4^+^ and γδ T cells and neutrophils in *Il10*^*−/−*^ vs. WT mice according to the manufacturer’s protocol. 30% of each sample volume was used for an isotype control. Data were acquired with a Fortessa FACS system (BD Biosciences) and analyzed with FlowJo. Doublets were excluded with FSC-A and FSC-H linearity.

### Immunohistochemistry and immunofluorescence

Immunofluorescence stainings for GFP and CD45 co-expressing cells were performed on 20 μm thick fresh frozen brain sections as previously described [22]. Cryostat cut sections were air-dried, rinsed in TBS for 10 min before blocked with 10% fetal calf serum in TBS + 0.5% Triton for 30 min at RT. Next, sections were incubated with rabbit anti-mouse GFP (ab290, Abcam) overnight at 4 °C. The following day the sections were rinsed for 10 min in TBS + 0.5% Triton before incubation with the cross-absorbed species-specific secondary antibody Alexa Flour 488 goat anti-rabbit (#A11070, Invitrogen) diluted 1:200 for 2 h. Next, the sections were rinsed in TBS for 10 min before incubation for 2 h with PE-conjugated rat anti-mouse CD45 (#553081 BD Biosciences). After a 2 × 10 min rinse in TBS the sections were transferred to TBS containing 40,6-diamidino-2-phenylindole (DAPI) (D1306, Invitrogen), rinsed in water, and mounted in gelvatol. Respective isotype controls were devoid of signal.

### Statistical analysis

Statistical analyses were performed using the appropriate test indicated in the figure legends. Briefly, the Student *t* test was used to compare infarct volumes, the Mann–Whitney *U* test for the comparison of clinical scores, and the one-way ANOVA test for multiple comparisons with the Bonferroni post hoc test. *P* values < 0.05 were considered statistically significant.

## Results

### IL-10 is protective following transient middle cerebral artery occlusion in mice

**Fig. 1:**
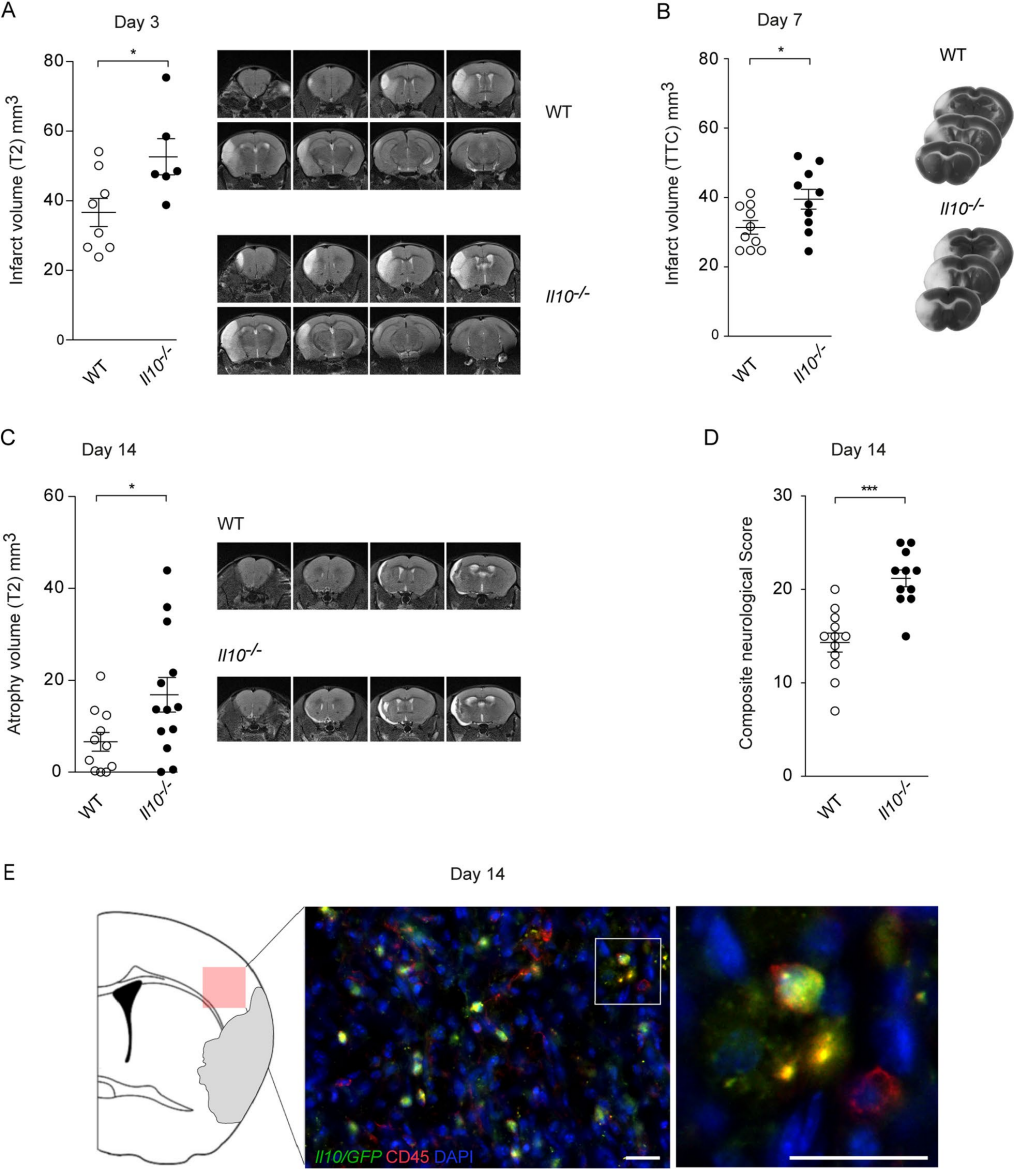
*Il10* deficiency leads to increased infarct sizes, enhanced brain atrophy, and poorer long-term outcome following tMCAO. MRI was used to quantify infarct volume at day 3 (**A**) and cortical atrophy volume at day 14 (**C**) after tMCAO in WT control and *Il10*^*−/−*^ mice (representative T2 image). **B** Triphenyltetrazolium chloride (TTC) staining was used for the evaluation of infarct volume at day 7 (representative TTC brain slices). **D** Composite neurological score was performed on day 14 after tMCAO. **E** *Il10/GFP*-positive cells were visualized in the penumbra area of the ischemic hemisphere by GFP counterstaining in *FIR-tiger* mice 14 days after tMCAO (green, *Il10/GFP*-positive cells; red, CD45-positive cells; blue, 40,6-diamidino-2-phenylindole nuclear staining; scale bar 20 µm). Infarct data are presented as mean ± SEM of 8–10 WT and 6–10 *Il10*^*−/−*^ mice (**A**, **B**), atrophy data as mean ± SEM of 11 WT and 13 *Il10*^*−/−*^ mice (**C**), and neurological score of 12 WT and 11 *Il10*^*−/−*^ mice (**D**). Statistical significances were analyzed by Student *t* test (**A**–**C**) and Mann–Whitney *U* test (**D**)*.* *P < 0.05 and ***P < 0.001

To investigate the role of IL-10 in acute ischemic brain damage and the subsequent recovery, we analyzed the neurological outcomes of Interleukin-10 knockout (*Il10*^*−/−*^) mice and wild type (WT) littermate controls up to 14 days after tMCAO. On day 3 and day 7 *Il10*^*−/−*^ mice exhibited significantly increased infarct volumes (Fig. 1A, B). Underlining the impact of IL-10 not only on the initial damage but also on the regeneration we found that *Il10*^*−/−*^ mice showed significantly increased brain atrophy and worse neurological scores on day 14 when compared to littermate controls (Fig. 1C, D, and Additional file 1: Fig. S1A). Mortality did not differ significantly between groups (Additional file 1: Fig. S1B).

### IL-10 is upregulated in infiltrating T cells and macrophages

To visualize the spatial distribution and cellular source of IL-10 in post-stroke brains, we performed immunohistochemistry using a reporter mouse expressing a green fluorescent protein (GFP) under the control of the IL-10 promotor. Fourteen days following tMCAO, we detected GFP-positive CD45^+^ cells in the penumbra area of the infarct indicating that immune cells contribute to the local IL-10 production in ischemic brains (Fig. 1E).

**Fig. 2:**
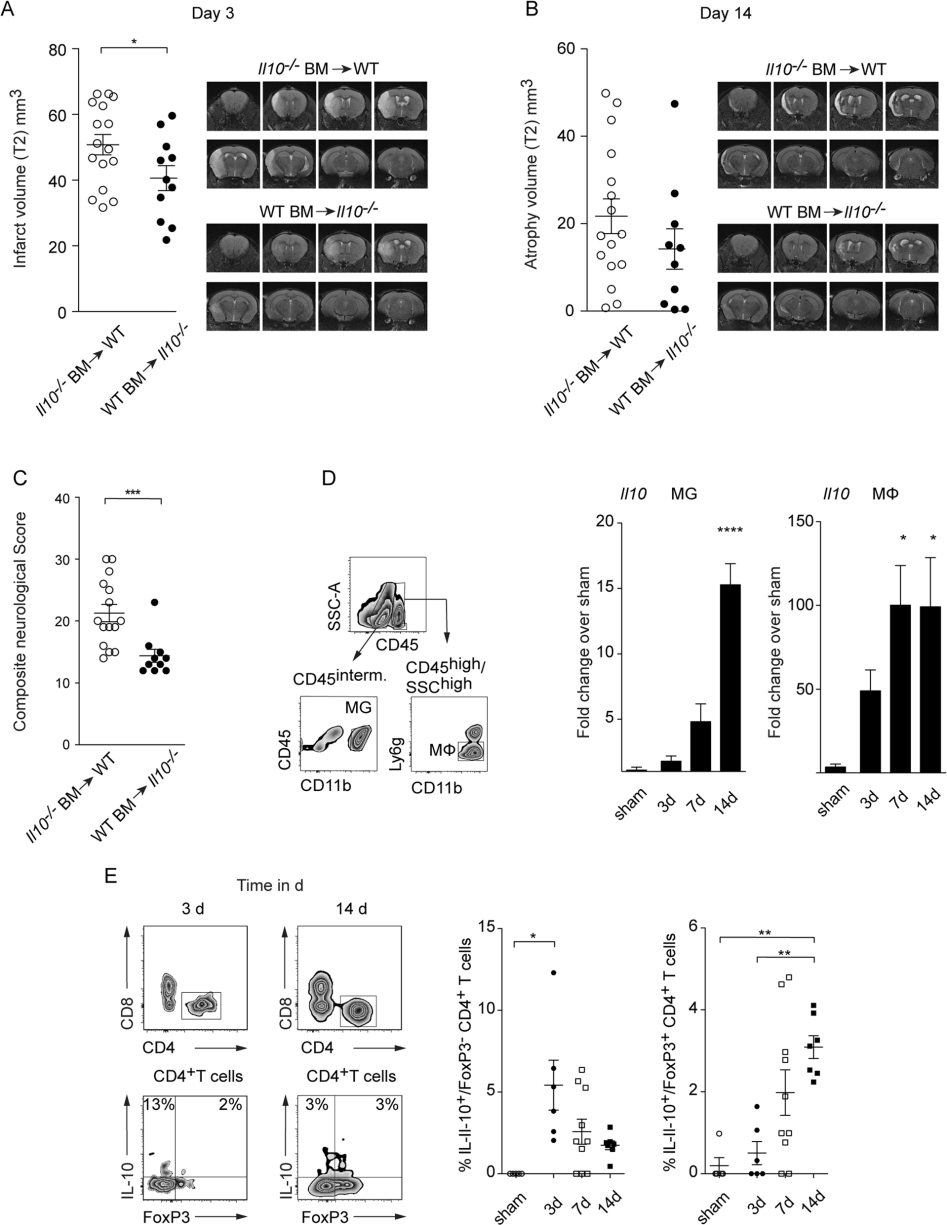
IL-10 from invading immune cells is neuroprotective. MRI was used to quantify infarct volume at day 3 and cortical atrophy volume at day 14 (**A**, **B**) after tMCAO in the chimeric mice (representative T2 image). **C** Composite neurological score was performed on day 14 after tMCAO. **D** Relative gene expression of *Il10* in brain resident CD45^intermed^/CD11b^+^ microglia and central nervous system-infiltrating CD45^high^/CD11b^+^/CD11c^−^/MHCII^−^/Ly6g^−^/F4/80^+^ macrophages purified 3, 7, and 14 days after tMCAO by fluorescence activated cell sorting from ischemic hemispheres of C57Bl/6 mice. Expression levels were normalized to corresponding levels of microglia after sham surgery or blood macrophages. **E** Flow cytometric analysis of IL-10 produced by CD4^+^ Tregs (Foxp3^+^) or non-Tregs (Foxp3^−^). Infarct and atrophy data are presented as mean ± SEM of 16 WT mice which received *Il10*^*−/−*^ and 10–11 *Il10*^*−/−*^ mice which received WT bone-marrow cells (**A**, **B**), neurological score as mean ± SEM of 15 WT mice which received *Il10*^*−/−*^ and 10 *Il10*^*−/−*^ mice which received WT bone-marrow cells (**C**), RT-qPCR gene expression data as mean ± SEM of 3–7 (**D**) and flow cytometric data as mean of ± SEM of 5–10 mice in each group (**E**). Significances analyzed by Student *t* test (**A**, **B**), Mann–Whitney *U* test (**C**), and 1-way ANOVA with Bonferroni post hoc test (**D**, **E**). *P < 0.05, **P < 0.01, ***P < 0.01 and ****P < 0.0001. *MΦ* macrophages, *MG* microglia

Next, we studied whether the IL-10 production from invading immune cells or brain resident cells including microglia, astrocytes, or neurons is decisive for the protective IL-10 effect following stroke. We generated bone-marrow chimeric mice using CD45.1^+^ WT or CD45.2^+^ *Il10*^*−/−*^ donor mice which were injected into CD45.2^+^ *Il10*^*−/−*^ or WT recipient mice, respectively. Following reconstitution > 98% leukocytes expressed congenic markers of the respective donor (Additional file 1: Fig. S2A, B). On day 3 after tMCAO we observed that *Il10*^*−/−*^ mice reconstituted with WT bone-marrow displayed significantly reduced infarct volumes when compared to WT littermate controls reconstituted with *Il10*^*−/−*^ bone-marrow cells. Underscoring the importance of IL-10 from peripheral immune cells, we further found that *Il10*^*−/−*^ mice reconstituted with WT bone-marrow displayed significantly improved neurological scores 14 days post-stroke, whereas brain atrophy did not differ significantly between groups (Fig. 2A–C).

To further investigate the temporal dynamics and the cellular source of IL-10, we measured mRNA levels of *Il10* transcripts in CD45^intermediate^, CD11b^high^ microglia and CD45^high^, CD11b^high^, Ly6g^negative^ macrophages on days 3, 7, and 14 post-stroke. Although contamination of macrophages with activated microglia is possible in this sorting strategy, it allows an estimation of IL-10 expression levels in both populations (Fig. 2D). Notably, macrophages showed significantly increased IL-10 level on days 7 and 14, whereas microglia showed a significant upregulation only on day 14, suggesting that the IL-10 production of macrophages and microglia is delayed following ischemia (Fig. 2D).

Besides microglia and macrophages, lymphocytes are an important source of IL-10. As the staining of IL-10 by hand is challenging we took advantage of the double-knock-in reporter *Fir-tiger* mouse model, which enables the simultaneous detection of IL-10 [green fluorescent protein (GFP)] and Foxp3 [monomeric red fluorescent protein (mRFP)] by flow cytometry. CD4^+^ T cells exhibited the highest frequency of IL-10 producers when compared to B and NK cells as well as CD8^+^ T cells (Additional file 1: Fig. S2C) [23]. Interestingly, in CD4^+^ T cells, we identified two different IL-10^+^ subpopulations that peak at different timepoints following stroke. On day 3 following tMCAO, IL-10^+^ Foxp3^−^ CD4^+^ T cells were significantly increased in frequency compared to sham surgery. Notably, these CD4^+^ T cells did not co-express the T_r_1 cell markers CD49b and Lag-3, while T_r_1 cells were detectable at the same timepoint in spleens of respective mice (Additional file 1: Fig. S2D) [23]. On day 7, the IL-10^+^ Foxp3^−^ CD4^+^ T cells decreased in frequency, while IL-10^+^ Foxp3^+^ Tregs appeared in ischemic hemispheres reaching significance on day 14 when compared to sham surgery (Fig. 2E).

Combined, this data suggests that the IL-10 production of infiltrating immune cells, in particular T cells and macrophages, is decisive for IL-10-mediated neuroprotection in stroke.

### IL-10 controls the IL-17A response in the inflamed brain

**Fig. 3:**
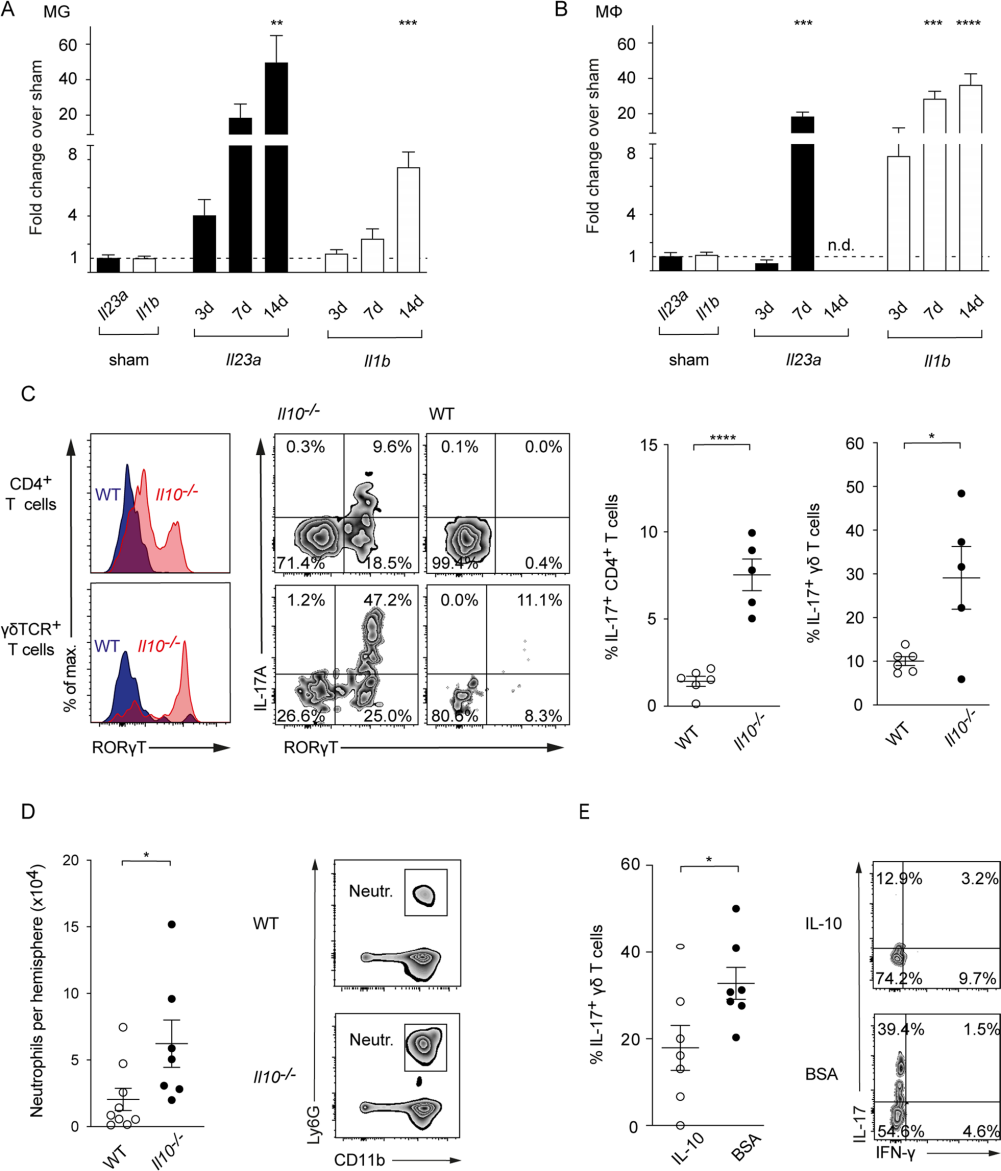
IL-10 controls the IL-17A axis in the proinflammatory milieu of the postischemic brain. Relative gene expression of *Il23a* and *Il1b* in brain resident CD45^intermed^/CD11b^+^ microglia (**A**) and central nervous system-infiltrating CD45^high^/CD11b^+^/CD11c^−^/MHCII^−^/Ly6g^−^/F4/80^+^ macrophages (**B**) purified 3, 7 and 14 days after tMCAO by fluorescence activated cell sorting from ischemic hemispheres. Expression levels were normalized to corresponding levels of splenic macrophages and microglia after sham operation. **C** Flow cytometric analysis of IL-17A produced by CD4^+^ and γδ T cells isolated from ischemic hemispheres of WT controls and *Il10*^*−/−*^ mice 7 days after tMCAO. **D** Flow cytometric analysis of number of infiltrating neutrophils in the ischemic hemispheres of WT controls and *Il10*^*−/−*^ mice 3 days after tMCAO. **E** Frequency of IL-17A producing γδ T cells purified from ischemic brains was analyzed 3 days following tMCAO in control mice (BSA) and mice receiving IL-10 intracerebral 3 h after tMCAO induction. **A**, **B** RT-qPCR gene expression data is represented as mean ± SEM of 4–7 WT, Flow cytometric data as mean ± SEM of 6 WT and 5 *Il10*^*−/−*^ (**C**), 9 WT and 7 *Il10*^*−/−*^ mice (**D**), and of 7 WT mice in each treatment group (**E**). Statistical significances were analyzed by one-way ANOVA with Bonferroni post hoc test (**A**, **B**) and by Student *t* test (**C**–**E**). *P < 0.05, **P < 0.01, ***P < 0.001, ****P < 0.0001

In models of autoimmunity including inflammatory bowel disease [8] or nephritis [9], IL-10 effects largely depend on the inhibition of IL-17A pathways. IL-17A-dependent inflammatory pathologies in turn are induced by the prototypical pro-inflammatory cytokines IL-1β and IL-23 among others. To investigate whether IL-1β and IL-23 are also upregulated following ischemia, we investigated their expression in microglia and macrophages sorted from ischemic hemispheres. In microglia, we observed a significant increase in *Il23a* and *Il1b* mRNA levels on day 14 (Fig. 3A). Macrophages showed a significant upregulation of *Il23a* and *Il1b* on day 7 and of *Il1b* on day 14, respectively (Fig. 3B). Overall, these results demonstrate a sustained upregulation of IL-17A-polarizing cytokines in the ischemic brain.

To investigate whether IL-10 is capable to control IL-17A pathways in the presence of IL-1β and IL-23, we next analyzed IL-17A level in αβ and γδ T cells from ischemic brains of *Il10*^−/−^ and WT mice. On day 7, we observed a significant increase in frequency and absolute counts of IL-17A^+^ CD4^+^ and IL-17A^+^ γδ T cells in the post-ischemic CNS of *Il10*^−/−^ mice when compared to WT littermate controls (Fig. 3C, and Additional file 1: Fig. S3A). Notably, in contrast to IL-17A positive γδ T cells, we detected almost no Th17 cells on day 7 in WT littermates, indicating that physiological IL-10 levels strictly suppress the IL-17A production in CD4^+^ T cells in ischemic brains.

The main IL-17A effect in the ischemic brain is the induction of neutrophil-attracting chemokines (e.g., CXCL-1) [4, 24] as well as Matrix Metalloproteinases [4, 25]. Consistent with increased IL-17A levels, we detected a significant upregulation of *Cxcl1* and *Mmp3* transcripts in the whole brain mRNA and a significant increase in absolute numbers of neutrophils at day 3 post-stroke in *Il10*^−/−^ mice, respectively (Fig. 3D, and Additional file 1: Fig. S3B).

Next, we performed intracerebral injections of 200 ng recombinant IL-10 or Bovine Serum Albumin (BSA) in WT mice 3 h following tMCAO to evaluate the capacity of IL-10 to suppress IL-17A production locally in the ischemic brain. Intracerebral IL-10 treatment significantly lowered the presence of IL-17A producing γδ T cells when compared to a BSA control 3 days post-tMCAO (Fig. 3E).

**Fig. 4:**
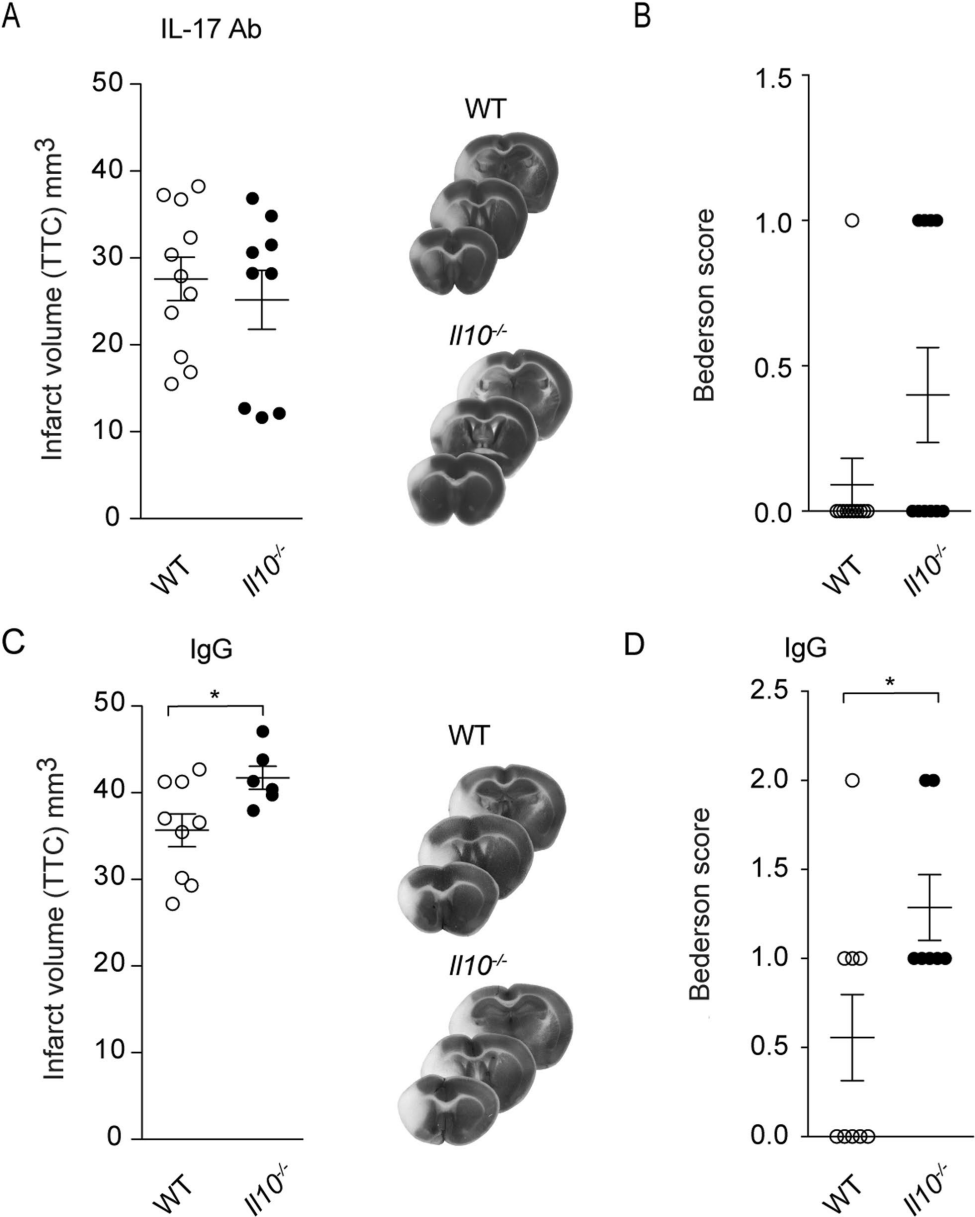
Neutralization of IL-17A abolishes the worse outcome of *Il10*^*−/−*^ mice. Triphenyltetrazolium chloride staining was used for evaluation of infarct volume at day 7 of IL-17A antibody (**A**) or IgG control (**C**) treated *Il10*^*−/−*^ and WT control mice. Bederson score was performed 7 days after tMCAO IL-17A antibody (**B**) or in IgG control (**D**) treated group. Infarct data are presented as mean ± SEM of 9–11 WT and 6–9 *Il10*^*−/−*^ mice per group. Bederson score as mean ± SEM of 9–11 WT and 7–10 *Il10*^*−/−*^ mice per group. Statistical significances were analyzed by Student *t* test (**A**, **C**) and Mann–Whitney *U* test (**B**, **D**). *P < 0.05

To show that the dysregulation of IL-17A is decisive for the worse outcome in *Il10*^*−/−*^ mice, we treated *Il10*^*−/−*^ mice and littermate controls with 500 µg IL-17A neutralizing antibody or IgG control 3 h and 3 days post-tMCAO. Anti-IL17A treatment rendered the infarct volume and the neurological scores of *Il10*^*−/−*^ and WT littermate controls almost equal 7 days post-tMCAO (Fig. 4A, B). Importantly, treatment with the IgG control antibody did not rescue the worse outcome of *Il10*^*−/−*^ when compared to littermate controls (Fig. 4C, D).

### *Differential effects of IL-10 on IL-17A*^+^*brain infiltrating CD4*^+^*and γδ T cells*

**Fig. 5:**
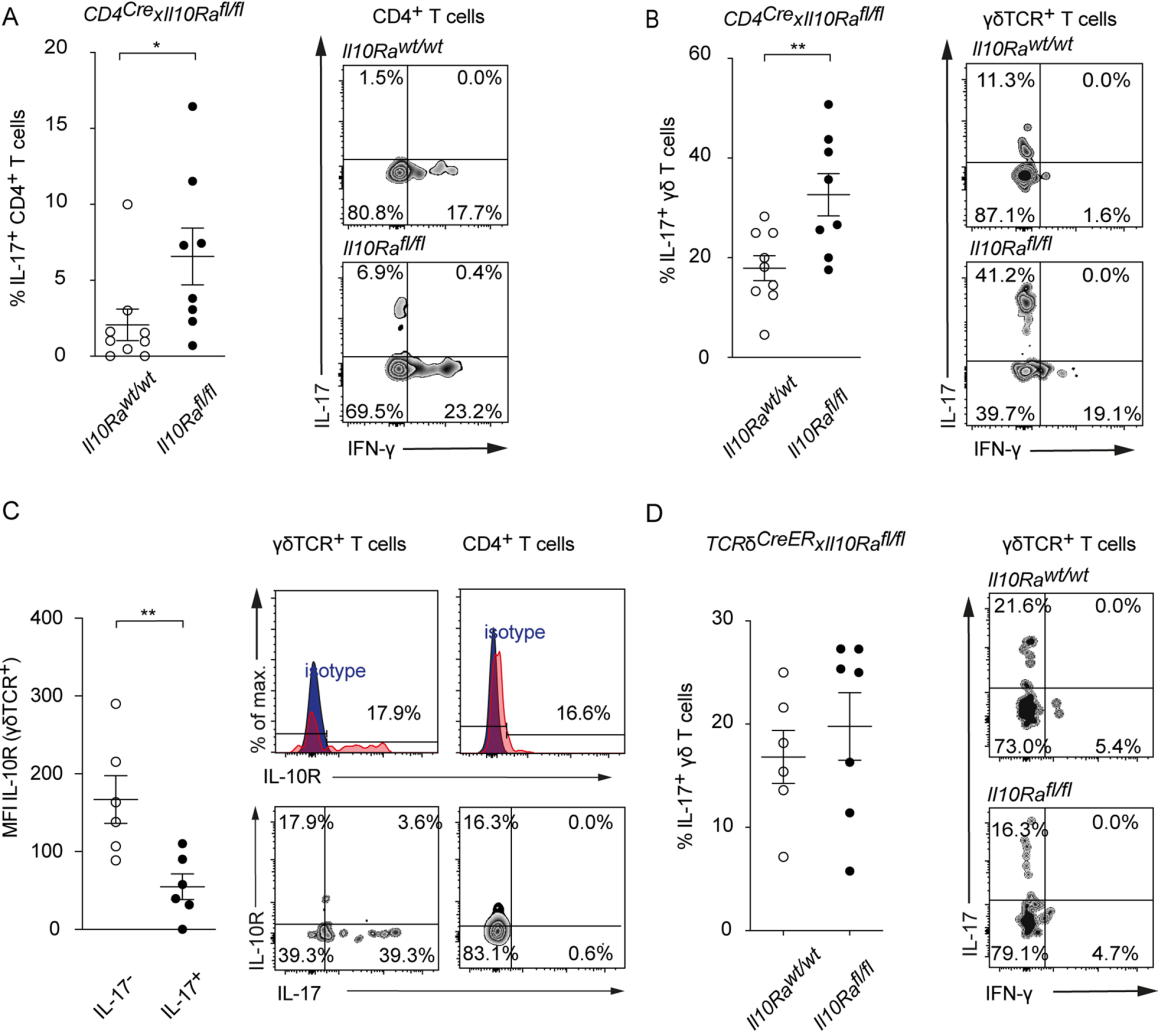
T cell-specific blockade of IL-10R leads to increased frequencies of IL-17A^+^ CD4^+^ and γδ T cells. Flow cytometric analysis of IL-17A produced by CD4^+^ (**A**) and γδ (**B**) T cells isolated from ischemic hemispheres of *CD4*^*Cre*^*Il10Ra*^*wt/wt*^ controls *and CD4*^*Cre*^*Il10Ra*^*fl/fl*^ 7 days after tMCAO. **C** Flow cytometric analysis of IL-10R (CD210) on IL-17A^+^ and IL-17A^−^ γδ T cells isolated from ischemic hemispheres of C57BL/6 mice 7 days after tMCAO. **D** Flow cytometric analysis of IL-17A produced by γδ T cells isolated from ischemic hemispheres of *TCR*δ^*CreER*^*Il10Ra*^*wt/wt*^ controls and *TCR*δ^*CreER*^*Il10Ra*^*fl/fl*^ 7 days after tMCAO. Flow cytometric data is represented as mean ± SEM of 9 *CD4*^*Cre*^*Il10Ra*^*wt/wt*^ and 8 *CD4*^*Cre*^*Il10Ra*^*fl/fl*^ (**A**, **B**), 6 WT (**C**) and 6 *TCR*δ^*CreER*^*Il10Ra*^*wt/wt*^ and 7 *TCR*δ^*CreER*^*Il10Ra*^*fl/fl*^ (**D**) mice per group. Statistical significances were analyzed by Student *t* test (**A**–**D**). *P < 0.05, **P < 0.01. *MFI* mean fluorescent intensity

To identify whether IL-10 directly inhibits the IL-17A response through the IL-10 receptor on T cells, we next employed transgenic mice, in which the IL-10 signaling is abrogated in mature CD4^+^ T cells. To generate a deletion of the α-chain of the IL-10R in CD4^+^ T cells which is essential for IL-10 signaling, we bred *CD4*^*Cre*^ with *Il10Ra*^*fl/fl*^ mice. We observed a significant increase in frequency of IL-17A^+^ brain-infiltrating CD4^+^ and γδ T cells of *CD4*^*Cre*^ × *Il10Ra*^*fl/fl*^ mice at day 7 following tMCAO when compared to *CD4*^*Cre*^ × *Il10Ra*^*wt/wt*^ control mice (Fig. 5A, B). As γδ T cells from *CD4*^*Cre*^ × *Il10Ra*^*fl/fl*^ mice have an intact IL-10 receptor, these data suggest that γδ T cells are inhibited through alternative pathways which are indirectly controlled by IL-10, whereas IL-10 exerts its inhibitory functions directly on CD4^+^ T cells.

Notably, IL-17A^+^ γδ T cells display a significantly lower IL-10R expression when compared to IL-17A^−^ γδ T cells. This finding further supports the hypothesis that IL-17A^+^ γδ T cells are not directly inhibited by IL-10 (Fig. 5C).

To confirm that IL-10 is not affecting γδ T cells directly, we bred *TCR*δ^*CreER*^ × *Il10Ra*^*fl/fl*^ mice. In line with our finding in *CD4*^*Cre*^ × *Il10Ra*^*fl/fl*^ mice, the selective depletion of the IL-10 receptor in γδ T cells had no direct effect on their IL-17A production (Fig. 5D).

In sum, these data suggest that CD4 T cells play a central role for the control of the detrimental IL-17A response in ischemic brains, and that the IL-10 signaling in CD4 T cells is essential for their protective function. That the targeted depletion of the IL-10 receptor on γδ T cells does not alter their IL-17A production, in turn, clearly indicates that the IL-17A production in these innate lymphocytes is not directly controlled by IL-10.

### IL-10R expression on Tregs is obligatory to inhibit IL-17A production in γδ T cells

**Fig. 6:**
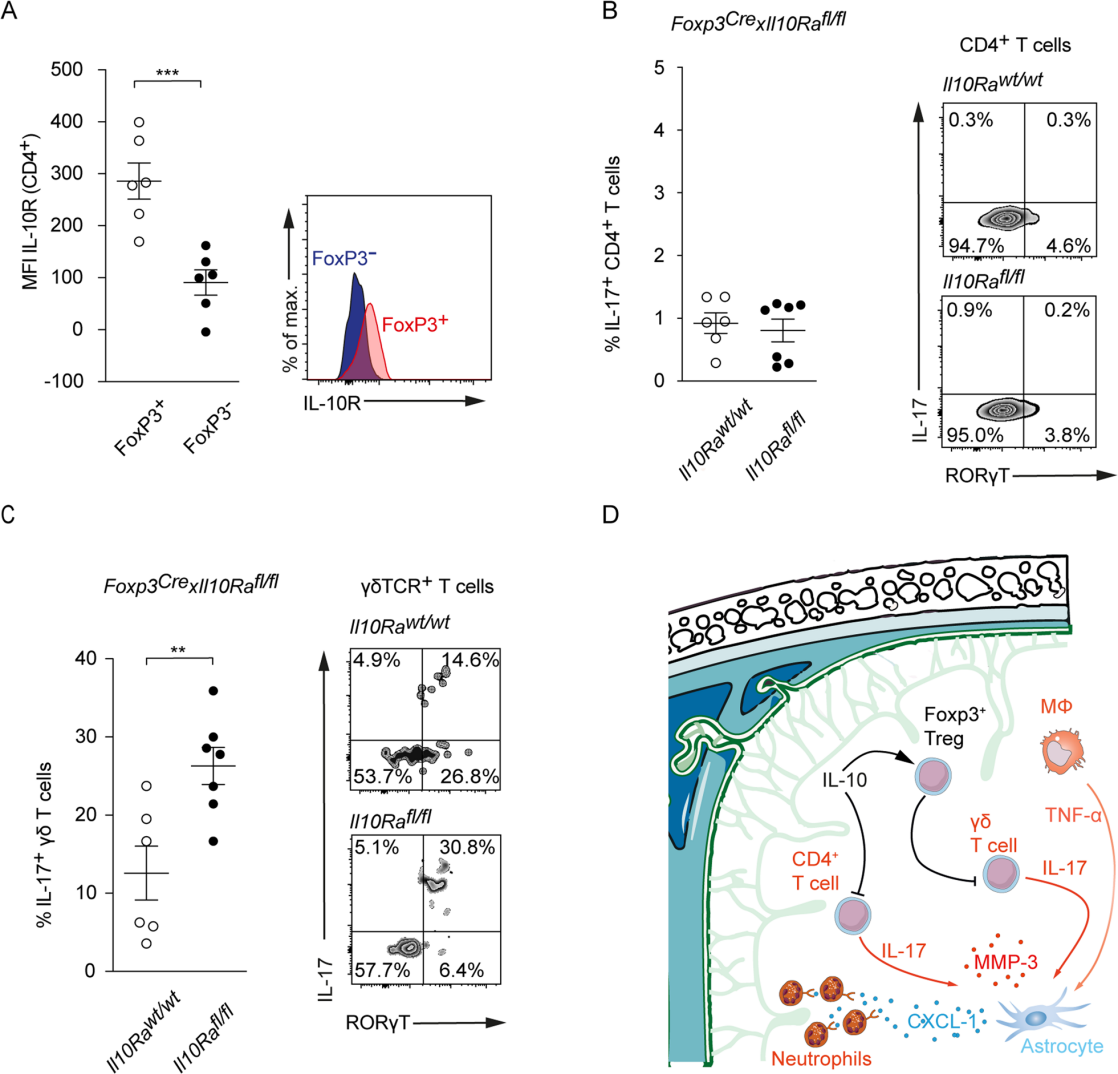
Control of IL-17A production in γδ but not CD4^+^ T cells is dependent on IL-10R expression on Foxp3^+^ Tregs. **A** Flow cytometric analysis of the IL-10R (CD210) Mean Fluorescent Intensity (MFI) on Foxp3^+^ and Foxp3^−^ CD4^+^ T cells in *FIR-tiger* mice. Flow cytometric analysis of IL-17A produced by CD4^+^ (**B**) and γδ (**C**) T cells isolated from ischemic hemispheres of *Foxp3*^*Cre*^*Il10Ra*^*wt/wt*^ and *Foxp3*^*Cre*^*Il10Ra*^*fl/fl*^ controls 7 days after tMCAO. (**D)** Proposed mechanism of protection from ischemic brain injury induced by IL-10. Flow cytometric data is presented as mean ± SEM of 6 *FIR-tiger* (**A**) and 6 *Foxp3*^*Cre*^*Il10Ra*^*wt/wt*^ and 7 *Foxp3*^*Cre*^*Il10Ra*^*fl/fl*^ mice per group (**B**, **C**). Statistical significances were analyzed by Student *t* test (**A**–**C**). **P < 0.01 and ***P < 0.01

To further investigate whether Treg or non-Treg CD4 T cells are capable to react to IL-10 and thus potentially control γδ T cells, we next analyzed expression levels of the IL-10 receptor in double reporter *Fir-tiger* mice. On day 7 following tMCAO, we observed a significantly higher expression of the IL-10 receptor in the Foxp3^+^ compared to the Foxp3^−^ compartment (Fig. 6A), further supporting that IL-10 can activate Tregs.

To further test whether Tregs are controlling γδ T cells in ischemic brains and if IL-10 confers Tregs the ability to inhibit the IL-17A production in γδ T cells, we bred *Foxp3*^*Cre*^ × *Il10Ra*^*fl/fl*^ mice to generate a Treg-selective deletion of the IL-10Rα. Seven days following tMCAO, we analyzed the frequency of IL-17A^+^ CD4 and γδ T cells derived from ischemic hemispheres (Fig. 6B, C). Upon deletion of the IL-10Rα in Tregs, CD4^+^ T cells displayed no skewing towards a Th17 phenotype. However, we observed a significantly increased IL-17A production in γδ T cells in *Foxp3*^*Cre*^ × *Il10Ra*^*fl/fl*^ mice (Fig. 6C).

It has been reported that programmed cell death protein 1 (PD-1) negatively regulates IL-17A levels in γδ T cells in a model of psoriatic-like skin inflammation [26]. To estimate the potential function of PD-1 in the control of IL-17A in γδ T cells in stroke, we analyzed PD-1 levels in γδ T cells from ischemic hemispheres and cervical lymph nodes. In line with a PD-1-dependent control of IL-17A^+^ γδ T cells we found a significantly increased frequency of PD-1/IL-17A double positive γδ T cells from ischemic hemispheres, when compared to PD-1/IL-17A positive γδ T cells from cervical lymph nodes (Additional file 1: Fig. S3C).

In summary, our data show that IL-10, which by itself had no direct effect on γδ T cells, is able to suppress the IL-17A production of γδ T cells via Tregs.

## Discussion

IL-17A is seen as a decisive factor for the early detrimental postischemic inflammatory response following stroke [3, 4]. One of the main effects of the IL-17A axis in ischemic brains is the induction of CXC-chemokines, which lead to a massive infiltration of neutrophils [4, 24]. However, in stroke, the initial IL-17A driven inflammatory response is short-lasting, indicating that the sterile inflammatory response is effectively controlled [4].

Mechanisms that limit the detrimental IL-17A response in stroke are not well understood. In this study, we tested whether the anti-inflammatory cytokine IL-10 controls the early IL-17A response in T cells following experimental stroke. We found that IL-10 expressed by invading leukocytes plays a key role in the regulation of the IL-17A production in both αβ and γδ T cells in ischemic hemispheres. Interestingly, the effects of IL-10 on CD4^+^ and γδ T cells differ. While IL-10 directly inhibits the IL-17A production in CD4^+^ T cells, signaling via the IL-10R endows Tregs with the capacity to downregulate IL-17A in γδ T cells. However, abrogation of direct IL-10R signaling in γδ T cells does not impact their IL-17A production, underlining that IL-10 controls the IL-17A production of αβ and γδ T cells differentially.

As a source of IL-10 we did not only identify Foxp3^+^ Tregs, macrophages and microglia in ischemic brains, but also Foxp3^−^ CD4^+^ T cells. Interestingly, these IL-10^+^/Foxp3^−^/CD4 ^+^ T cells were negative for CD49d and Lag-3, indicating that these are not T_r_1 cells. Given the plasticity of T cells under inflammatory conditions, it is possible that the Foxp3^−^/CD4^+^ / IL-10^+^ T cells are T_h_1 [27], T_h_2 [28] or even T_h_17 [29] cells that participate in the resolution of inflammation.

Furthermore, it should be considered that other infiltrating immune cells and resident cells such as B cells [30] and astrocytes [31] are additional sources of IL-10, which endow Tregs to control the IL-17A production in γδ T cells. The role of IL-10 from brain resident cells is supported by our data from bone-marrow chimeric mice. Although bone-marrow chimeras with an IL-10-deficient peripheral immune compartment displayed significantly larger infarcts at 3 days following tMCAO, when compared to bone-marrow chimeras with an IL-10-deficiency in brain resident cells, brain atrophy was not significantly altered at 14 days between both groups. This observation indicates that IL-10 from peripheral immune cells is involved in the control of the initial damaging inflammatory response, whereas IL-10 from resident brain cells might have trophic functions during regeneration.

IL-10 has been implicated in several studies to provide neuroprotection in the brain [6, 32]. However, in stroke, only Benakis et al. found that IL-10 is protective through the control of IL-17A pathways [24]. The authors showed that the modulation of the gut microbiome diminished the frequency of IL-17A^+^ γδ T cells in the gut in an IL-10-dependent manner which was associated with an attenuation of the IL-17A driven postischemic inflammatory reaction in the brain. Beyond these observations, our data indicate that IL-10 not only controls the IL-17A driven postischemic inflammatory response in the peripheral immune compartment but also directly in the ischemic brain. We showed that intracerebral injections of recombinant IL-10 reduce the frequency of IL-17A producing γδ T cells in the ischemic brain following tMCAO.

A strict regulation of IL-17A level in the CNS may also have physiological functions. Recent studies showed that IL-17A^+^ γδ T cells populate the meninges from perinatal stages to build a self-renewing pool which controls neuronal signaling, behavior [33], and cognition [34] under homeostatic conditions. The fact that IL-17A can have important homeostatic functions in the brain on the one hand and be proinflammatory on the other hand is explained by the physiology of IL-17A signaling. Outcomes of the IL-17A receptor activation depend on the cooperative stimulation by other cytokines, such as IL-1β and TNF-α [35], which are upregulated in the ischemic brain tissue and can lead to synergistic activation of downstream signaling pathways [22]. IL-17A alone is only a modest activator of inflammatory pathways.

During post stroke inflammation, IL-10 seems to directly inhibit the IL-17A production in Th17 cells in ischemic brains, which is a known mechanism from other models of autoimmunity [8]. Accordingly, the targeted deletion of the IL-10R in CD4^+^ T cells leads to increased frequencies of Th17 cells in ischemic brains. In contrast to Th17 cells, IL-10 does not directly control IL-17A^+^ γδ T cells. Signaling via the IL-10R rather endows Tregs with the capacity to downregulate IL-17A in γδ T cells. In this study, we did not identify the molecular factor, which enables IL-10R^+^ Tregs to inhibit IL-17A^+^ γδ T cells. Possible candidates include the B- and T-lymphocyte attenuator and the programmed cell death protein 1 as it has been reported that they negatively regulate IL-17A in a model of psoriatic-like skin inflammation [26, 36]. A possible role of inhibitory PD-1 signaling to control IL-17A^+^ γδ T cells in stroke is supported by our data showing an increased frequency of PD-1^+^ IL-17A^+^ γδ T cells in the ischemic brain when compared to cervical lymph nodes.

Translating findings from animal models to human patients is still challenging. However, we previously showed in autoptic brain tissue that the expression of IL-17A in T cells goes along with a massive neutrophil infiltration, indicating that the IL-17A–neutrophil axis contributes to human pathophysiology as well [4]. Regarding the potent and pleiotropic immunosuppressive properties of IL-10 on the systemic immune compartment, IL-10 itself might not represent an ideal therapeutic target in stroke. Stroke patients have a significantly increased susceptibility for pneumonia due to aspirations and an immunosuppressive state [37]. Our data show that the worse outcome in *Il10*^*−/−*^ mice is largely driven by increased IL-17A levels in the brain, which can be counteracted via neutralization of IL-17A. With this in mind, a targeted antagonization of IL-17A seems promising, irrespective of individual IL-10 levels pre-stroke. Besides, several experimental studies have already shown that the neutralization of IL-17A is protective in stroke [3, 4, 24]. Together with inflammasome activation [38], IL-17A is another conserved signal that orchestrates the amplification of the inflammatory response. In previous studies we observed increased IL-17A levels in ischemic brains as early as 6 h post stroke [4]. In contrast, the migration of neutrophils into the ischemic brain parenchyma is a comparatively late event during post-stroke inflammation, reaching its maximum at day 3 [4]. Therefore, to effectively target the development of the damaging immune response before it has amplified, anti-inflammatory therapeutic interventions should be given as early as possible. In this respect, it is possible that the therapeutic time window of an IL-17A-neutralization is even below that of thrombolysis, which is currently 6 h. IL-17A antibodies are already approved for the treatment of psoriasis [39] and severe adverse effects on the immune status have, thus far, not been apparent. However, prior to a clinical application of IL-17A antibodies in stroke, long-term data on the effects of IL-17A neutralization in both sexes should be collected to adjust for potential negative effects. This is necessary to exclude that the neutralization has negative long-term consequences on post-stroke regeneration by inhibiting the recently discovered beneficial IL-17A effects on memory and behavior [33, 34].

## Conclusion

In conclusion, our study provides additional insights into the complex interplay between different T cell subpopulations during post-stroke neuroinflammation. We have shown that IL-10 is pivotal for the control of pathogenic IL-17A^+^ αβ and γδ T cells and that an excessive IL-17A production is harmful. Thus, our study improves the understanding of the IL-17A-dependent postischemic inflammatory response and may pave the way towards new immunomodulatory therapies for stroke treatment.

## Supplementary Information

**Additional file 1: Figure S1.** *Il10*^*−/−*^ mice exhibit worsened neurological scores compared to WT littermate controls, whereas mortality did not differ between groups. (A) Bederson score and survival rate; (B) 14 days after tMCAO in WT control and *Il10*^*−/−*^ mice. **Figure S2.** Generation of bone-marrow chimeric mice and characterization of post-ischemic IL-10 producers. (A) Generation of bone-marrow chimeras. (B) Representative FACS plots of peripheral blood of bone-marrow chimeras after sacrifice. (C) Flow cytometric analysis of IL-10 produced by B cells, NK cells, CD8^+^, and CD4^+^ T cells after sham surgery and 3, 7, and 14 days after stroke in *FIR-tiger* mice. (D) Expression of Tr1 cell markers CD49b and Lag-3 on CD4^+^ T cells in spleen and CNS 3 days after tMCAO in *FIR-tiger* mice. **Figure S3.** IL-10 deficiency leads to an activation of the IL-17A axis and IL-17A positive γδ T cellls co-express PD-1 in the post ischemic brain. (A) Flow cytometric analysis of the number of infiltrating IL-17A^+^ CD4^+^ and γδ T cells isolated from ischemic hemispheres of WT controls and *Il10*^*−/−*^ mice 7 days after tMCAO. (B) Relative gene expression of *Cxcl1* and *Mmp3* in ischemic hemispheres of WT and *Il10*^*−/−*^ mice 3 days after tMCAO. (C) Flow cytometric analysis of IL-17 production and PD-1 co-expression by γδ T cells in cervical lymph nodes and the CNS 3 days after stroke.

## Data Availability

The data sets used and analyzed during the current study are available from the corresponding author on reasonable request.

## Abbreviations

IL-17A: Interleukin-17A
tMCAO: Transient middle cerebral artery occlusion
IL-10: Interleukin-10
IL-10R: Interleukin-10-receptor
Tregs: Regulatory T cells
T_R_1: T regulatory type 1 cells
WT: Wild type
BSA: Bovine serum albumin
MRI: Magnetic resonance imaging
*Il10*^*−*^^*/*^^*−*^: Interleukin-10 knockout
*FIR*: *Foxp3-IRES-mRFP*
*tiger*: *Il10-IRES-GFP-enhanced reporter*
CXCL-1: C-X-C Motif Chemokine ligand 1
MMP-3: Matrix Metalloproteinase-3
PD-1: Programmed cell death protein 1
