# Supplementary material for: Interleukin-10 improves stroke outcome by controlling the detrimental Interleukin-17A response
Source: J Neuroinflammation. 2021 Nov 13;18:265. doi: 10.1186/s12974-021-02316-7 (PMC8590298; doi:10.1186/s12974-021-02316-7)
Supplement: Supplementary file 1 — Additional file 1: Figure S1. Il10−/− mice exhibit worsened neurological scores compared to WT littermate controls, whereas mortality did not differ between groups. (A) Bederson score and survival rate; (B) 14 days after tMCAO in WT control and Il10−/− mice. Figure S2. Generation of bone-marrow chimeric mice and characterization of post-ischemic IL-10 producers. (A) Generation of bone-marrow chimeras. (B) Representative FACS plots of peripheral blood of bone-marrow chimeras after sacrifice. (C) Flow cytometric analysis of IL-10 produced by B cells, NK cells, CD8+, and CD4+ T cells after sham surgery and 3, 7, and 14 days after stroke in FIR-tiger mice. (D) Expression of Tr1 cell markers CD49b and Lag-3 on CD4+ T cells in spleen and CNS 3 days after tMCAO in FIR-tiger mice. Figure S3. IL-10 deficiency leads to an activation of the IL-17A axis and IL-17A positive γδ T cellls co-express PD-1 in the post ischemic brain. (A) Flow cytometric analysis of the number of infiltrating IL-17A+ CD4+ and γδ T cells isolated from ischemic hemispheres of WT controls and Il10−/− mice 7 days after tMCAO. (B) Relative gene expression of Cxcl1 and Mmp3 in ischemic hemispheres of WT and Il10−/− mice 3 days after tMCAO. (C) Flow cytometric analysis of IL-17 production and PD-1 co-expression by γδ T cells in cervical lymph nodes and the CNS 3 days after stroke. [file 12974_2021_2316_MOESM1_ESM.docx]

**Additional file 1**

**Interleukin-10 improves stroke outcome by controlling the detrimental Interleukin-17A response**

Marius Piepke, Bettina H. Clausen, Peter Ludewig, Jonas H. Vienhues, Tanja Bedke, Ehsan Javidi, Björn Rissiek, Larissa Jank, Leonie Brockmann, Inga Sandrock, Karoline Degenhardt, Alina Jander, Vanessa Roth, Ines S. Schädlich, Immo Prinz, Richard A. Flavell, Yasushi Kobayashi, Thomas Renné, Christian Gerloff, Samuel Huber, Tim Magnus and Mathias Gelderblom


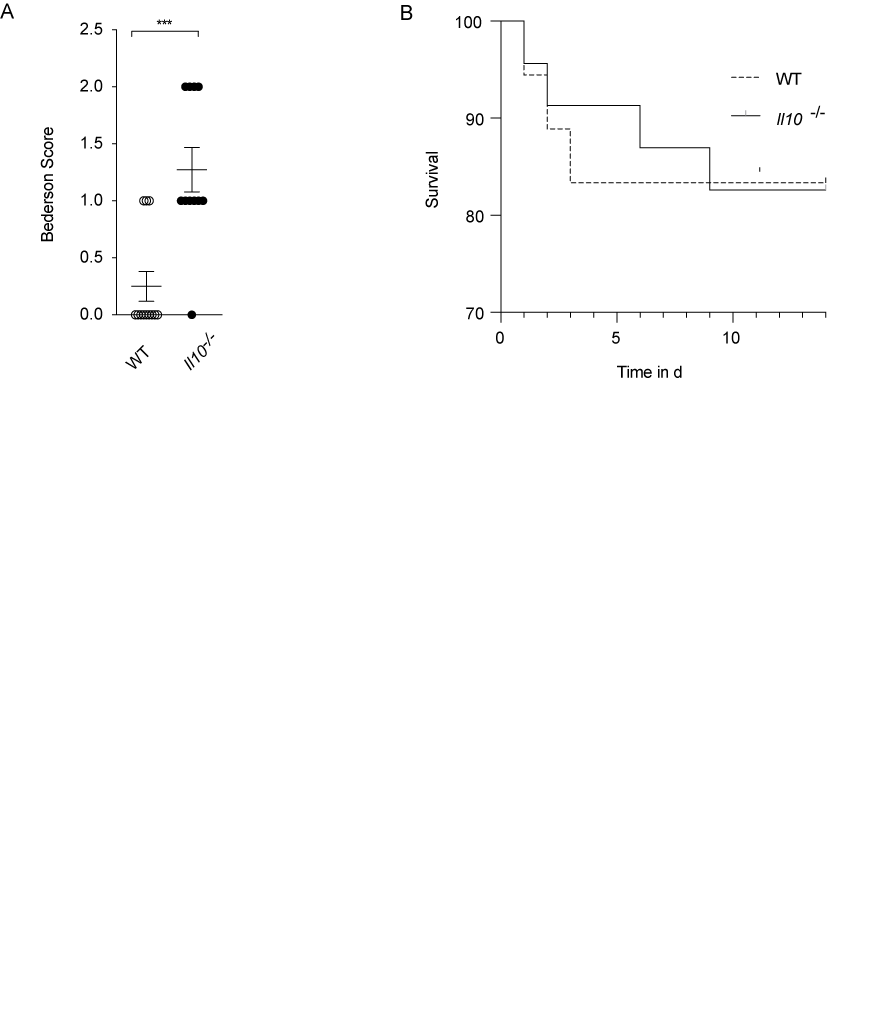


**Figure S1**

***Il10^-/-^* mice exhibit worsened neurological scores compared to WT littermate controls whereas mortality did not differ between groups**

Neurological score (Bederson) (A) and survival rate (B) 14 days after tMCAO in WT control and *Il10^-/-^* mice. Neurological score as mean ±SEM of 12 WT and 11 *Il10^-/-^* and survival data presented as mean ±SEM of 19 WT and 22 *Il10^-/-^* mice. Statistical significances were analyzed by Mann-Whitney *U* test and χ2 test (survival rate)*.* ***P<0.001.

**
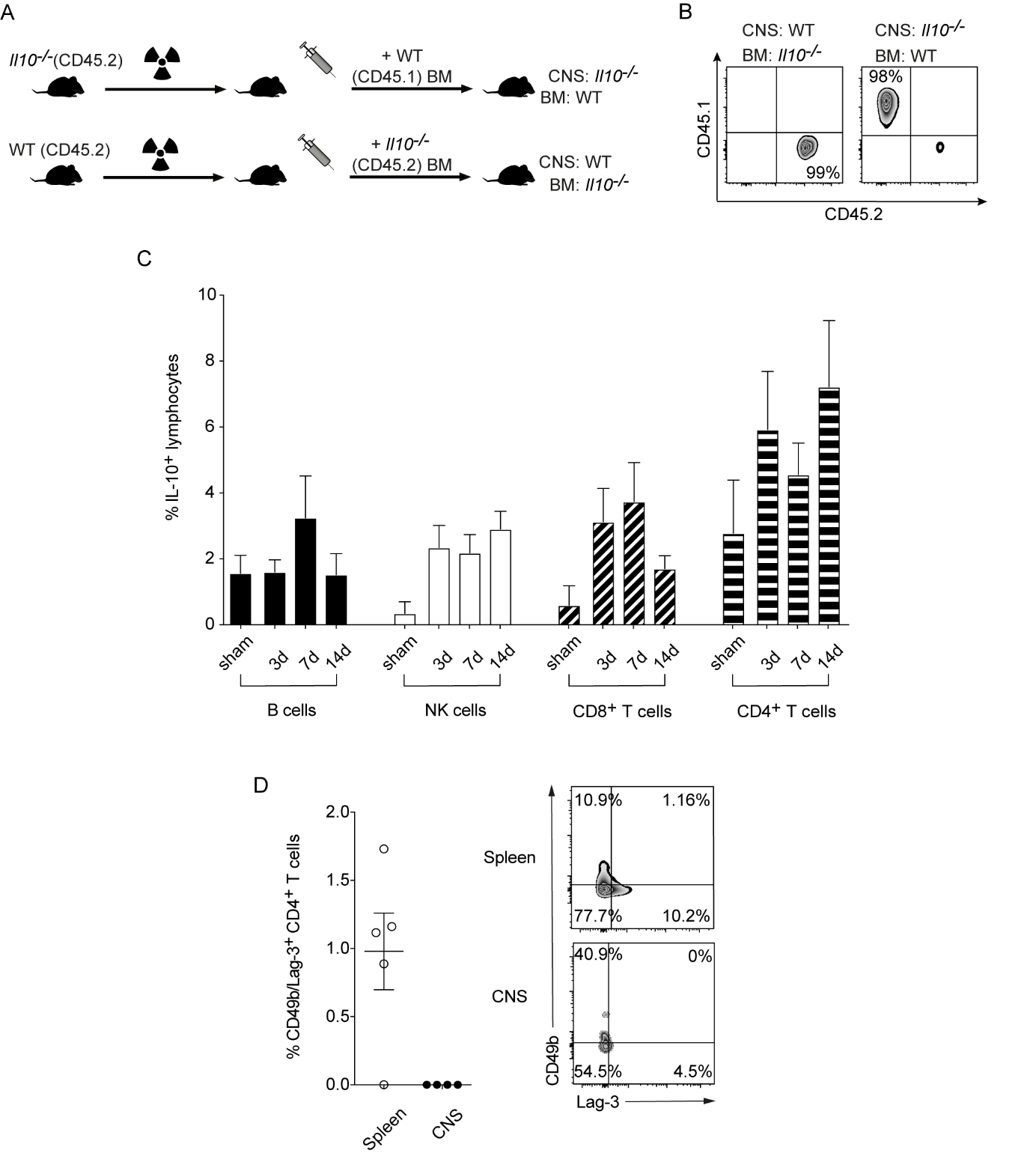
**

**Figure S2**

**Generation of bone marrow chimeric mice and characterization of post-ischemic IL-10 producers**

(A) Generation of bone marrow chimeras. (B) Representative FACS plots of peripheral blood after sacrifice. (C) Flow cytometric analysis of IL-10 produced by B-cells, NK-cells, CD8^+^, and CD4^+^ T cells after sham surgery and 3, 7, and 14 days after stroke in *FIR-tiger* mice. (D) Expression of Tr1 cell markers CD49b and Lag-3 on CD4^+^ T cells in spleen and CNS 3 days after tMCAO in *FIR-tiger* mice. Flow cytometric data is presented as mean ±SEM of 4-10 mice per group.


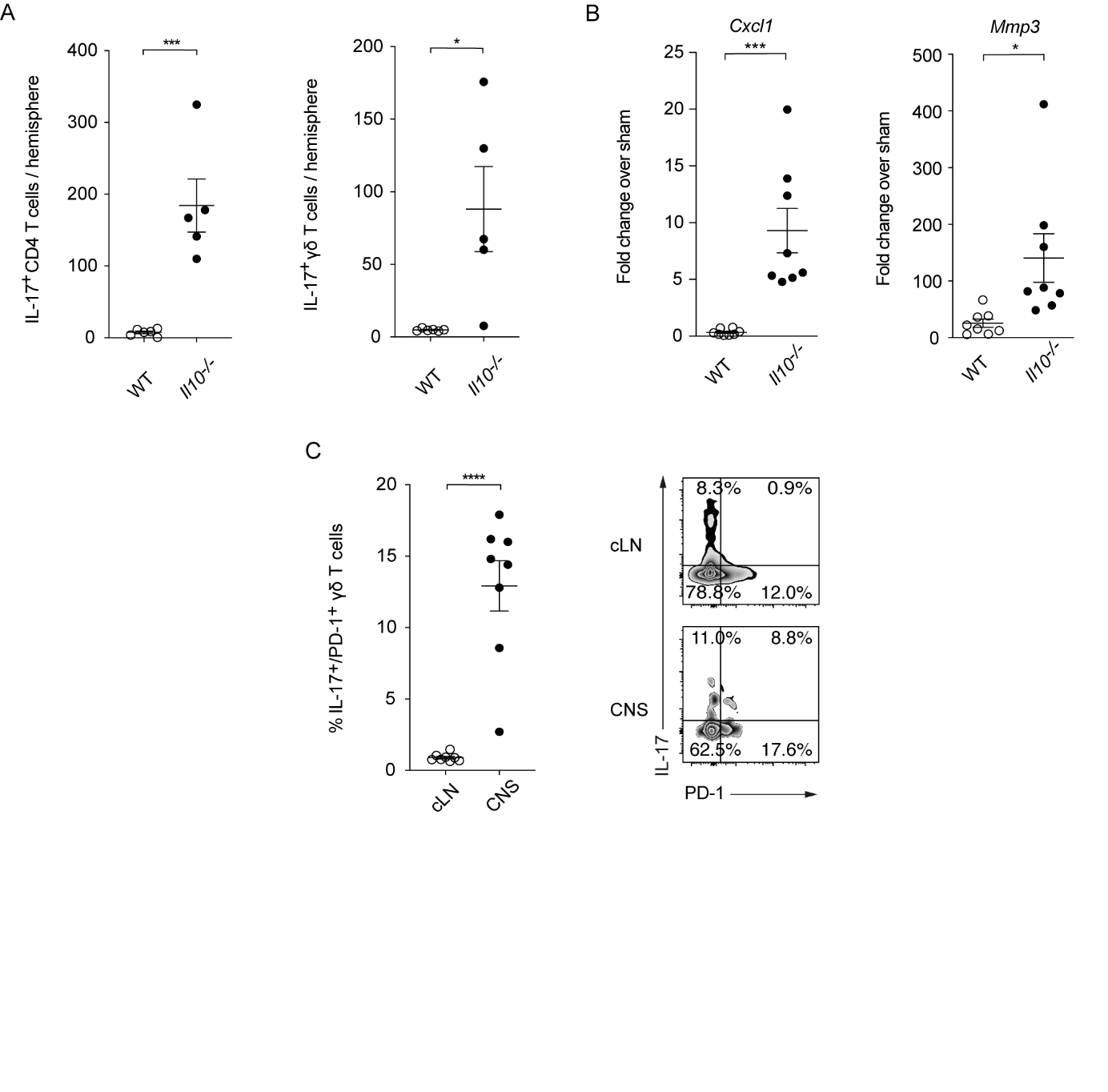


**Figure S3**

**IL-10 deficiency leads to an activation of the IL-17A axis and IL-17A positive γδ T cellls co-express PD-1 in the post ischemic brain**

(A) Flow cytometric analysis of the number of infiltrating IL-17A^+^ CD4^+^ and γδ T cells isolated from ischemic hemispheres of WT controls and *Il10^−/−^* mice 7 days after tMCAO (B) Relative gene expression of *Cxcl1* and *Mmp3* in ischemic hemispheres of WT and *Il10^−/−^* mice 3 days after tMCAO. (C) Flow cytometric analysis of IL-17 production and PD-1 co-expression by γδ T cells in cervical lymph nodes (cLN) and the CNS 3 days after stroke. Flow cytometric data is presented as mean ±SEM of 5-8 mice per group (A and C), and RT-qPCR gene expression data as mean ±SEM of 8 WT and *8 Il10^-/-^* mice per group (B). Statistical significances were analyzed by Student t test (A-C). *P<0.05, ***P<0.001, ****P<0.0001.
